# The incurable metastatic breast cancer experience through metaphors: the fight and the unveiling

**DOI:** 10.1080/17482631.2021.1971597

**Published:** 2021-08-28

**Authors:** Alexandra Guité-Verret, Melanie Vachon

**Affiliations:** a Psychology Department, Université Du Québec À Montréal, Montréal, Canada; b Center for Research and Intervention on Suicide, Ethical Issues and End-of-Life Practices, Montreal, Canada; c Réseau Québécois De Recherche En Soins Palliatifs Et De Fin De Vie (Rqspal), Quebec, Canada

**Keywords:** Metastatic breast cancer, metaphor, blog, war metaphor, cancer experience, suffering, authenticity, end-of-life, interpretative phenomenological analysis

## Abstract

**Purpose:** War metaphors are omnipresent in public and medical discourse on cancer . If some studies suggest that cancer patients may view their experiences as afight, few studies focus on the metaphors that patients create from their subjective experiences. The aim was to better understand the experience of four women with incurabale metastatic breast cancer from the metaphors they used in personal cancer blogs.**Methods:** An interpretive phenomenological analysis (IPA) was used to analyze these women's experience and metaphors of cancer.**Results:** Two metaphors carried the meaning of metastatic breast cancer experience: the fight and the unveiling. The results show that the war metaphor had a unique meaning for the bloggers who lived with incurable breast cancer: they revealed the difficulty of fighting cancer and eventually collapsing in battle, although a renewed look at life had developed in parallel to their struggle. The bloggers thus tried to lift the veil on this complex experience.**Conclusion:** The results highlight the need for women with metastatic breast cancer to be able to tell and share their experience in a supportive context and to reinvest the war metaphor in order to express themselves in a more authentic way.

## Introduction

Breast cancer is the most common cancer worldwide (World health Organization (WHO), 2020). Among women in Canada, it accounts for about a quarter of new cancer diagnosis per year (Brenner et al., 2020). Although there has been an important increase in life expectancy for women affected by the disease in recent decades, about 30% of women develop metastatic breast cancer (MBC), which is generally incurable. Also known as stage 4 breast cancer or advanced breast cancer, MBC is a cancer that starts in the breast, spreads to one or more other parts of the body, and cannot be definitively eliminated. Due to biomedical advances, however, women with MBC may live through several years with treatments (chemotherapy, radiotherapy, hormone therapy) to reduce symptoms and control the progression of the disease (Thorne et al., 2013). The desire to live longer may motivate a maximization of treatments, which would nevertheless imply a major and exhausting commitment on the part of these women (García‐Rueda et al., 2016). The side effects of life-prolonging treatments, accompanied by the effects of cancer, would in all probabilities cause a lot of suffering considering for example, the state of disability in which these women may find themselves and the uncertainty surrounding their death (García‐Rueda et al., 2016; Vilhauer, 2008).

It must be said that cancer affects all aspects of a patient’s life, altering the relationship to oneself, to his/her body, to others and to the world (Marin, 2014). The disease often raises existential questions about life and death and existential distress, which can lead to a search for meaning (Best et al., 2015; Mosher et al., 2013; Yalom, 1980). To the extent that the diagnosis of incurable cancer requires the transition from curative to palliative medicine, women with MBC are particularly aware of their mortality. These women may then seek to live a life which matches their own personal values (Sarenmalm et al., 2009) and develop a renewed appreciation for life (Willis et al., 2015). However this quest must consider the reality of a daily life in which they face a decline in their physical and social activities (Mosher et al., 2013). A recent systematic review of qualitative evidence (Smit et al., 2019) shows that women’s experiences with breast cancer are defined by great fatigue, body limitation, existential distress, vulnerability, and a need for support. Yet women with MBC often suffer from a lack of support from their loved ones and feel isolated, especially because they have difficulty or hesitate to open up to them about their suffering (Ginter, 2020), and because they fear affecting their well-being by acknowledging the dark side of the disease (Krigel, 2014). As a result a significant part of their experience remains silent (Mayer & Grober, 2006).

Because their experience is so far removed from the socially constructed narrative about cancer, namely that of “survivors” who have triumphed over cancer (Willig, 2011), women with MBS often remain silent. In public discourse on cancer and specifically on breast cancer, the war metaphor is omnipresent (Nie et al., 2016), as evidenced by the broad approaches several countries and organizations take “to fight cancer”. This metaphor reflects the Western socio-medical imperative demand to win the “war against the disease” (Mino et al., 2008) and against death itself (Callahan, 2017). According to Segal (2012) and Nielsen (2019), there is a standard narrative about breast cancer that promotes certain values or ways of living the disease: being strong in the face of disease, fighting and surviving, being positive and optimistic. These are all values inscribed in breast cancer culture, symbolized by the colour pink (Segal, 2012). Several authors suggest that these values can discourage the expression of contrary experiences and leave aside the existential dimensions of the disease such as consciousness of death and lack of meaning (Hurley, 2014; Vitry, 2010). This would be even more problematic for patients with incurable cancer, as the process of finding meaning is based precisely on the integration of death (Willig, 2009). Strength and positivity, linked to the image of combat, would contradict the experience of many women with MBC, who report having little control over life (Lewis et al., 2016; Vilhauer, 2008) and live under the shadow of death (Sarenmalm et al., 2009).

While the use of the war metaphor in this field has been criticized by many doctors and researchers (Harrigton, 2012; Oronsky et al., 2016), and while its use may contradict the experience of incurable cancer, there are still very few empirical studies that examine the metaphors that patients use or create (Hui et al., 2018; Southall, 2013). Some studies suggest that patients with incurable cancer are imbued with warlike images, but that they also approach the disease from the metaphors of journey, imprisonment or burden (Hommerberg et al., 2020; Semino et al., 2018). However these studies are based on quantitative evidence and do not look at the lived experience of patients in the light of the metaphors they use. Moreover, no qualitative study has yet looked at the metaphors used by women with MBC, although breast cancer has unique existential and cultural issues.

For several years, many women with MBC have been speaking on social networks and sharing their experiences through elaborate stories (Baik et al., 2019). Among various virtual spaces, the blog, because of its intimate nature, is particularly useful for expressing the experience of illness and suffering (McCosker, 2008). The distinctive feature of the blog is to give the blogger space to put into words a personal narrative that unfolds through time. One may think that its use, for many women, counterbalances isolation and social injunction to remain silent. Storytelling can facilitate the process of finding meaning, as the disease is transformed into a coherent narrative that can be shared with others and integrated into one’s personal life story (Martino & Freda, 2016). Writing means building a story when illness tends to deconstruct and undo everything: foundations, bonds, friendships (Marin & Sarthou-Lajus, 2008, p. 49).

### Aims

Considering the problem surrounding war metaphors in the literature on breast cancer and the lack of research on the metaphorical expression of women themselves, and given the richness of the blog as a space for expression, the objective of the present study was to better understand the experience of women with MBC from the metaphors they use on their blog. The study asks this question: what does the experience of cancer mean for women with MBC, as revealed by their metaphors?

### Theoretical and methodological approaches

This explorative study followed the approach and guidelines of Interpretative Phenomenological Analysis (IPA) (Smith et al., 2009). The theoretical underpinning of IPA comes from phenomenology and hermeneutics (Smith, 2018). IPA “is phenomenological in attempting to get as close as possible to the personal experience of the participant, but recognizes that this inevitably becomes an interpretative endeavor” for the researcher (Smith et al., 2009, p. 37). In this view, IPA assumes phenomenological work is always an interpretative process, that is a hermeneutic phenomenology.

A basic understanding of phenomenological research is that the meaning of a phenomenon—here incurable breast cancer—is created between subject and object, between researcher and data (Ponterotto, 2005; Smith et al., 2009; Vinit, 2016). Through this creative “encounter”, researchers must be transparent and sincere about their methodological assumptions, while acknowledging their subjectivity during the process of understanding (Tracy, 2010; Willig, 2019). This belongs to another basic understanding of phenomenology: the need for researchers to “bracket”—to put aside—the taken-for-granted world in order to concentrate on their perception of the participants’ world (Smith et al., 2009, p. 15). Bracketing however can only be partially achieved because researchers always bring their prior experiences, assumptions and preconceptions to the encounter (Smith et al., 2009). Therefore, IPA connects bracketing with reflexive practices. For this study, we saw and dealt with our own process of perception and understanding by keeping a reflexive journal and by discussing with peers about the research.

IPA approach is referred to as a “double hermeneutic” in which the researcher interprets the way the participant interprets his/her lived experience (Tuffour, 2017). As part of this study, the first hermeneutic related to the experience of incurable breast cancer as express by each woman on her blog, especially through metaphors. The second hermeneutic corresponded to our interpretation of their narratives and metaphors. For IPA, the way researchers interpret the data and the way the participants interpret their own experience depend on the same mental skills but still remain different because the researcher’s interpretation is second order and employs those skills in a more self-conscious, systematic way (Smith et al., 2009). Throughout this interpretative process, we followed Tracy’s (2010) criteria of quality in qualitative research, including rigour, sincerity, credibility and ethics.

Finally, this study refers to metaphors as defined by phenomenological philosophy rather than by linguistic. In this view, a metaphor is a fundamental path of experience and a search for meaning (Ricoeur, 1991; Tuffour, 2017). Metaphors produce a deviation in the usual use of words, destroy one order to create another, which leads to a re-description of reality and an unprecedented relationship to the life world (Ricoeur, 2003).

## Method

### Material

The study sample consisted of four blogs about the experience of incurable breast cancer. This small and homogeneous sample served as an in-depth analysis of this particular phenomenon (Smith et al., 2009). Blogs were collected in 2020 on the basis of the following inclusion criteria:

- The blogs had to be written by a woman with incurable MBC and not living in a care institution.
- The blogs had to be written in French or English.
- The blogs had to consist of complex descriptions of illness.
- The blogs had to be inscribed in Western culture.
- The blogs had to be accessible to the public.

The selected blogs were written by four women who had been diagnosed with stage 1 to 3 breast cancer in the past. As a result, these women had already experienced curable breast cancer and possibly remission at the time of being diagnosed with incurable MBC. In addition, all the women, exept for one, began to write their blog from the moment they received the MBC diagnosis. We analysed the stories from the moment the patients received this final diagnosis. Table I provides some characteristics of the sample.

**Table I.** Blogs characteristics

	Blogger’s name	Nationality	Blog’s writing period	Blogger’s age when diagnosed with metastatic breast cancer
**Blog 1**	Marie	Canada	2019–2020	35
**Blog 2**	Julie	United-States	2011–2019	53
**Blog 3**	Céline	France	2013–2020	42
**Blog 4**	Audrey	United-States	2012–2020	46

### Data analysis

Interpretive phenomenological analysis (IPA) (Antoine & Smith, 2017; Smith & Osborn, 2003; Tuffour, 2017) is a flexible and dynamic process rather than a standardized rigid method. First, the principal investigator conducted an immersive reading of each story individually and annotated on the margins potentially significant excerpts in relation to the woman’s experience. This first step was followed by a second and new reading, this time to identify the units of meaning and metaphors of each narrative, taking care to remain anchored in the very words of each woman.

The units of meaning and the metaphors of the stories were then organized and put into dialogue in order to identify the metaphorical themes emerging. At this stage, it was a matter of identifying the meaning of experience from a global existential perspective by asking the following question: “For this woman, in this context, what does this metaphor mean and what does it tell us about the experience of breast cancer?” From this stage resulted two metaphorical themes, divided and condensed into nine sub-themes.

Finally the narratives and the reflexive notes were re-read in order to deepen our understanding of the two metaphorical themes and to verify the correspondence between these themes and each woman’s narrative. This last step led to condense the expression of the two essential metaphorical themes into five final sub-themes.

### Ethical considerations

This study received ethical board approval from the university where the research took place (Montreal, Canada). Since consent from the bloggers was not needed because the blogs are public material, the integrity of all bloggers was protected and fictitious names were used so as not to reveal their identities. We also assumed ethical considerations continue beyond the data collection phase to how researchers interpret human suffering. In practice, we prioritized (1) a posture of humility in the face of death and illness, (2) an openness to otherness, and (3) an awareness of the universality of finitude (Bourgeois-Guérin & Beaudoin, 2016; Vachon, 2019).

## Results

The interpretive phenomenological analysis of the blog narratives allowed the identification of two metaphorical themes that carried the meaning of incurable breast cancer experience: (1) the fight, and (2) the unveiling. In this section, each of the two metaphorical themes will be further analysed, illustrated through blogger quotes.

### The fight

Our understanding suggests that all four women lived and understood the incurable disease as a complex and violent-like journey. Their narratives revealed the difficulty of fighting cancer and eventually collapsing in battle, although a renewed look at life can developed at the very heart of this struggle.

#### The paradox at the heart of the fight

All four bloggers conceived their experience from the war metaphor, which was a way of understanding both the medical path they followed and the aim of the treatments doctors prescribed them. The framing of the illness experience in terms of a fight was however paradoxical. On the one hand, the women used the image of combat to express their desire to live, to “continue” with life, especially in the first months after their fatal diagnosis. On the other hand, they distanced themselves from this fight against cancer. Indeed they seemed to be less the initiators of the fight than the receptacle embodied of the fight against cancer conducted by doctors. Although palliative, the treatments recommended by doctors were often seen as “chemical weapons” serving the “offensive” against cancer. In addition, the paradoxical use of the war metaphor in the blogs rested on the central place of death in the cancer experience. In fact the women were engaging the fight even though it was “lost in advance”. Facing the horizon of death, this fight was matter of medical failure for these women but even more, it was a sign of a personal failure experienced daily:

I have become aware that this is a fight that I am destined to lose. […] It is extremely difficult to live your life on chemo, your body a continual battleground against the enemy, especially one who is merely yourself. I know that someday, I will get too tired. […]. But not yet. I fight on. (Julie)

On Friday, I will meet with my doctor to devise a plan of attack. I’m not sure what the plan will entail, but I’m guessing a little bit of everything … surgery, chemo, radiation, drugs … not necessarily in that order. But I know that I am ready to give it all I have got. I like being alive, I enjoy being here and I will fight to stay as long as possible. I know I have a long road ahead, but I also believe in myself and my strength. […] Cancer may end up winning the war … In the end … but I have MANY battles I plan on winning along the way. (Audrey)

In addition to the lost battle against the disease, it appears to us there was a fight against oneself. The women questioned the meaning of their medical journey. Because the cancer was incurable, their suffering caused by treatments often appeared senseless. In this context, the repetitive nature of the treatments undergone initiated a fight against oneself. Right after a consultation with the oncologist, Céline related the following:

I’m afraid of the violence of this new attack. I’m afraid our chemical weapons are not strong enough to retaliate. I’m afraid I’m not willing to fight anymore. So far I have always thought that treating cancer is not a courageous fight, but an imposed necessity. […] Here I am less categorical all of a sudden. I feel that when the cancer lasts, when it invites itself in successive salvos, when one thinks that it has been pushed back enough to have respite […], it comes back again and again and harder … So yes, it’s a fight. A hell of a fight even, but a fight against oneself and the desire to give up. For it takes a lot of courage, yes, to go back to the front knowing the battle is lost in advance. (Céline)

Our analysis suggests that this double struggle led by the women was linked to a deeper paradox: while they sought to chase away death to have more time with their loved ones, they felt their suffering was increasingly pervasive and that they were more and more “disconnected” from the common world. From this perspective, the possibility for the women to stop the fight against cancer, in other words to stop treatments, seemed to refer as much to a personal defeat in relation to the medical programme, as to the discomfort of being half alive, like a “lifeless creature”.

Among the bloggers, only Marie went so far as to claim to want to stop treatments. Unlike the treatments that were “endured” because “there are no other choices”, the cessation of the fight meant the possibility to choose; that is, making a choice against all outside influences:

After several weeks of reflection, of having isolated myself from everyone, so as not to be influenced, I decided to stop fighting. For me to live is not to survive. I know many of you will judge me. That many of you would have tried everything, all possible treatments to try to win this war against cancer. But I’m bowing out. I don’t have a quality of life anymore and it’s never going to come back the way it used to be. I am losing my autonomy because I am constantly dizzy, in a wheelchair for long trips, I just sleep and I gradually become a wreck for my family (even if they all tell me it’s not so!). […] It’s time for me to let go and let life run its course. (Marie)

To all four women, the idea of stopping treatments seemed to correspond to the adoption of a passive posture in relation to the disease and more broadly in relation to life. “Let life take its course”, “abandon the fight”, “let yourself be carried away by events”, “let go” … The blogs are full of these less combative phrases in which the women found a meaningful expression of their experience. This passive attitude was consistent with the progressive loss of ability to act that they described. The adoption of such a position implied, however, a separation from the every-day world in which fighting was necessary. Accepting what life has in reserve for them was not always a guarantee of reconciliation with others.

#### Collapsing in battle

The stories of the women also told of the devastation the treatments inflict on their bodies and the significant diminishing opportunities for action that this entailed for them. When the women felt “tired” and “powerless” in the face of cancer or felt so “exhausted” they could not participate in the most ordinary activities, they seemed to relate the extent of a personal collapse. The women perceived these treatments as a violent force directed towards themselves as well as against the cancer. Indeed, despite prolonging their lives, the treatments were the source of great suffering and led to the acceleration of life towards old age and death:

Radiation therapy on my lungs saved my life. I don’t look down on it. But finally, if it could have come by my house without vandalizing everything, I would have been grateful. […] I’m cured. Yippee. However my insides are burnt, shriveled, diminished, ravaged for life. (Céline)

Immediately after infusion last week, I came home, went to bed and stayed in bed until Sunday. No steroid energy. Just bread, nausea, vomiting, weakness and illness. Chemo came at me in a fury, like a 500 pound Sumo wrestler determined on giving me the beat-down of my life. […] After seven chemos, the air is going out of me, my skin is wrinkling … (Julie)

It largely seemed that the collapse experienced by the women is a metaphoric downward movement; that is, a decrease in the sense of self. This collapse reflected a loss of power over disease and life. The women could no longer projected themselves in the world and in the future, because the horizon of possibilities was greatly reduced, whether it concerned projects, decisions, relationships, jobs. The collapse could even gave rise to a loss of identity. Indeed the women no longer seemed to recognize themselves, diminished as they were compared to what they were “before cancer”:

Metastasized breast cancer, don’t fool with it. It will have the last word, no matter what anyone says to reassure you. It invited itself, it will stay. It is at home, everywhere and nowhere at once, ready to appear when it has decided. So make yourself small so you don’t wake up the angry one. You’re lying low. You’re flattened out … You’re not fighting, no way. You’re not brave either. You obey the doctor. You ingest without flinching. (Céline)

I have faced the reality of my disease but it is much harder to face the reality of my decline. (Julie)

The whole of this dark truth usually jumps in my face when a small detail rekindles what I once was, illuminating in doing so, the stunted life of today and what is to come. (Céline)

The experience of self-diminution could be soothed by the benevolent presence of others (family, friends, caregivers) and their support. This human contact may have enabled the women to give new meaning to their disease:

You see, people with cancer experience some major “lows”. […] But I truly believe that having others to walk the road alongside us, lifts us up. (Audrey)

#### A renewed look

Our understanding suggest that all four bloggers conceptualized the cancer experience as having two sides, one violent, and the other brighter. After the announcement of incurable cancer, the women seemed to begin to take a new look at their lives. One is led to think that the awareness of death encouraged them to renew their understanding and meaning of life, its fragility, its value and flavor. Death then could become meaningful, triggering a change of values, choices and ethical positioning in relation to one’s existence. This very clear view of their own existence was well expressed within the metaphor of the photographic “focus”. It would seem, therefore, that death opened the way to a “renewed” and more authentic appreciation of life:

I did a tune-up and I had to do a big clean up in my values and re-prioritize them. I think we should all take a moment to clean up our lives, we shouldn’t wait for an ordeal to force us to do it. (Marie)

Well it’s not cancer that’s a great friend, dear reader. I knew that, though. No, it’s what it triggers. The big kick in the back. The correction for the eyes too, to see better, frontways, which is important. The decision to finally accomplish what has been postponed for too long. The departure. The transition from empty to real life. From the despair of certain death, to the happiness of life certainly well lived. (Céline)

Knowing your life is short makes you think deeply and acknowledge your mortality in a vivid way. It forces you to put things into perspective, makes you want to cut out the noise to get to the Song. (Julie)

While the women seemed to be fighting in vain and felt the world closing in on them, the opportunity to live a more authentic life represented a new opening to the world. It became a matter of “capturing” life and investing it as an opportunity, a power, but also as a knowledge that they could bequeath to others through the narrative of their lives. This renewal, part of a violent narrative, allowed them to find meaning in the face of illness and death.

### The unveiling

Through the blogs, the women sought to reveal the “hidden” face of their incurable cancer experience, a portrait of a complex struggle they will not be able to win. It allowed them to develop a sufficiently honest narrative about their experience. Our analysis suggests that the women tried to lift the veil on the suffering experienced and the complexity of their incurable disease.

#### Lifting the veil on suffering

For all bloggers, death appeared to be the horizon from which they understood their daily sufferings, their current possibilities of action, and the new meanings that life could take. However, the blog narratives revealed the women’s difficulty to talk about death outside the blog space. In that way, the women had to deal with the lack of a place, outside the blog, in which they could be heard. Therefore an important part of their experience remained unknown to those surrounding them. They sometimes had no choice but to hide their emotions within, by putting on “the mask of the strong woman”, by “hiding her rage” or by “crying in silence”. This idea of a silent experience seemed to go hand in hand with the isolation felt in relation to the cancer experience. The women’s suffering would often remain silent until it was identified on the blog and incorporated into each person’s life story:

I, unfortunately, am a realistic person, who does not even believe in God, not even something after death. For me death is the end, there’s nothing, it’s black, it’s over! And I know that thinking like that sounds negative. But I can’t help it. These thoughts I keep to myself, I do not want to contaminate the morale of others around me who cling to miraculous stories they have heard to keep hope. (Marie)

I’m crying, of course. But I cry with my chin up, for my men, big and small. So yes I’m crying! Oh not at home, of course. Not in sight of those I love, those who matter to me and who rely on me. I only cry with the doctors. That’s the rule. And even then: when I feel the emotion swells up, when it gets bigger, when it grows and grows, I grit my teeth, I lock the valves very quickly. They let the first tears through, and then hold back everything else. I cry because I can’t get rid of it. I cry because I’m sick … It’s been going on for centuries … […] I cry because it’s bound to end too soon. (Céline)

As shown in these quotes, the women often stopped themselves from expressing the full range of their emotions associated with cancer (fear of death, anguish, anger, sadness, despair) to avoid “disturbing” others. This tendency to exclude their loved ones from their suffering indicated a difficulty to express their emotions, but also an ethical concern for others. As a result one must then found in oneself a place to preserve the essence of the lived experience.

If the women kept some facets of the disease to themselves, other facets did not have to be hidden, being already invisible to the world:

I hate the degraded face I see in the mirror. Puffy, crumpled, pleated, dry, without eyelashes or eyebrows. The short, sparse hair. The pale moon face, extinguished. So much ugliness, so much silent and invisible suffering. (Céline)

The non-recognition of the suffering had important consequences on the ability of the women to share their experience and to accept sensitive support from others. We have noted among the women the presence of a tension between their need to restrain their suffering in order to not show it in the open and, at the same time, the regret that this suffering escaped the eyes of others. The blog narrative would nevertheless help to expose the silent experiences, as writing meant building a shared and meaningful narrative about suffering. Thus the metaphor of the unveiling would be a sign of openness to oneself, to others, to the world and to the meaning of the disease:

I decided to write this blog as a way to express myself and better understand my emotions. I’m not someone who opens up easily to others and I hate to talk about my emotions because I feel it makes me look weak. (Marie)

Our analysis points out that the women who had been living with incurable cancer for several years were affected by not meeting certain medical standards related to cancer, for example, when they had not lost their hair or when they were no longer undergoing typical cancer treatments. For these women, silent suffering was articulated differently but led to isolation similar to that of other women. For Julie, the isolation took on the appearance of a bitter “secret”:

We are dying, yet we don’t need to park in the handicapped area; we don’t look sick, we wait in line, we listen to people talk about their small aches and pains and petty problems, and we are standing behind them with this big huge secret. (Julie)

#### Lifting the lid on the complexity of incurable cancer

If, for all bloggers, the path of the disease seemed to represent a linear road (which leads to death), this route was not free of irregularities and oscillations, more like the “ups and downs” of a roller coaster. The women refused to cast a veil over the experience of cancer, which consisted both of moments of deep anguish when they felt oppressed and downtrodden, in the “depths” of the disease, and moments of elevation when they felt uplifted by joy, hope or the harmonious relationship to others. It seemed to us that some women created the metaphor of oscillation to detach themselves from an overly positive portrait of cancer or from an overly strong representation of themselves. Indeed, the writing of the blog reflected the need to draw a clear distinction between an insincere optimism and an authentic consciousness of death. And yet, the women were making every effort to moderate the narrative of the “negative” or “bad” dimensions of their experience, whether by apologizing in advance to readers or by detailing the efforts they have put into being positive:

I sometimes struggle writing this blog. Cancer is a ride that takes me through many twists and turns, ups and downs, highs and lows. Part of me wants to always be positive, to look at the good and downplay the bad. At the same time, it wouldn’t be honest to do that. (Audrey)

I know that this text may not be as positive as the last ones … But I want to portray my reality as I live it with its ups and downs. […] I stay strong (most of the time) and I try to get through this ordeal as best I can. I have no other choice! I wanted by this text to tell you that I am not a superwoman, I am human and imperfect. (Marie)

So, that’s the update from cancerland. Not the most uplifting, but I always try to balance optimism and reality. Right now, I’m drowning in reality. (Audrey)

Even on their blog the women suppressed part of their lived experience, perhaps because they associated the expression of vulnerability with a lack of strength or perseverance. However, this unequivocally positive posture was difficult to maintain because of the complexity of the disease and the personal collapse that they attached to their new identity. Although the women did gave a place to the “high” moments of the disease, the heart of their stories was about the “lows” of the disease. This composition of their narratives appeared essential in order to reveal the reality of self-collapse, despite the struggle.

Through the metaphor of oscillation, the bloggers could better integrate uncertainty in relation to the unpredictable evolution of the cancer. The movement between the top and the bottom would then be understood and perceived as an oscillation between life and death:

All of us with metastatic breast cancer understand that. We are given a chemo that works, shrinks our tumors. We get weeks, or months of time where we feel okay, hey the stuff is working! […] the next scan shows the drugs have stopped working and the tumors are growing. Our hopes are dashed again - we are going to die after all. So, your doctor tries a new one, which may, or may not, work. But either way, it won’t work for long. We are on the up and down rollercoaster of mets. (Julie)

We are mourning our lives while living them, existing in the shadow between life and death, all the while wondering how long until the final chemo stops working. (Julie)

Life can be sweet and beautiful … and interspersed with booster stitches, sometimes I’ll forget I’m sick. (Céline)

The complexity of incurable breast cancer was also revealed by its fundamental difference with breast cancer leading to remission. The women felt isolated on the “dark” trajectory of metastatic breast cancer, while breast cancer was described as broadly part of a “pink” culture whose trajectory excludes death and abandons all patients with fatal prognosis. Feeling the burden of the dire MBC prognosis, the women began to reflect on a scale no longer personal and familial but collective. Aware of how difficult it could be for the healthy to comprehend the question of dying, they felt the need to relate, on the blog, their long and painful departure from the everyday world; that is, writing the blog was an attempt to communicate to the alter world the reality of their suffering. In this context, the blog was an important space in that it revealed metastatic breast cancer as a unique, devalued, unrecognized and socially marginalized disease:

I realized when I told my story that it is a disease that is very little known to most people. We prefer to talk about beautiful stories of women who have beaten stage 1 to 3 breast cancer […]. But unfortunately, the statistics bring us back to a darker reality … (Marie)

Would you appreciate being excluded from the road of those who are still being treated? If it happened to you, you wouldn’t say something like “That’s it, I’m being pushed out … . to the gaping hole that awaits me!” On the contrary, we need to feel that we are not forgotten. (Céline)

Those of us who are metastatic don’t fit into the pink narrative as promoted by major charities and which has become part of our culture. […] We metastatic patients are the losers. […]. Those of us who actually face death are often treated as pariahs and as outsiders—we haven’t toed the pink line. (Julie)

The above narratives tell us this incurable disease could not be articulated in the dominant narrative of cancer, as if one were evolving in parallel with the other. This discrepancy led the women to feel they have been forgotten, which accentuated the silence that was already imposed on them. They maintained their experience, as painful as it is, deserves to be recognized as a possible horizon of breast cancer, despite the failure it can represent in our contemporary societies. More broadly, the blogs call for the recognition of metastatic breast cancer as a possible horizon of life.

## Discussion

This study sought to better understand the experience of four women with incurable MBCs using their online stories and the metaphors they contain. Our analysis suggests that these women viewed the cancer experience as a fight, difficult to disclose because it was a lost battle from the outset. In fact, the stories invoked a dynamic of veiling-unveiling that paced and reinvested this common issue of battle: the bloggers described their experience as a lost fight against the disease and a fight against oneself.

Being researchers in the hermeneutic phenomenology tradition, we suggest that every individual is caught in a social and spoken world that precedes him/her, and that it is from this world that every individual interprets his/her reality (Tuffour, 2017). Our study indicates that the women used and integrated their cancer experience from the war metaphor, so often used in our contemporary societies to understand disease and death. More important, our study reveals a new aspect of the war metaphor when breast cancer is incurable. Unlike the war metaphor, usually linked to a triumphant survival that sidelines the issue of death, the war metaphor used by the women with MBCs represents the other side of the fight, since it expresses a sense of failure and an increased awareness of death. This sense of personal failure seemed to reflect the medical failure associated with their condition; it tinges the way they integrate their finitude. Our analysis points to this fight as being fought with less vigour by the women themselves than by medicine (doctors, treatments). This may reflect a lack of decision-making and action opportunities, the result of a “spectator” posture. This posture fits the multiple restrictions often experienced in this context, whether in terms of control over life and commitment to life (Lewis et al., 2016; Vilhauer, 2008; Willis et al., 2015), or in terms of decision-making in the trajectory of care (Lavoie & Dumond, 2015).

The war metaphor was also consistent with the metaphor of collapse. Both allow the women to describe and understand their sense of being beset by treatments and diminished by disease. To “decline”, to “fall down” became an altered mode of being-to-the-world. For example, chronic pain and fatigue represented changes to the biological body but also to the “lived body” (Merleau-Ponty, 1945), as pain and fatigue limited the women in their subjectivity and contact with the world. The collapse refers to an experience of loss and separation—loss of participation in joint activities, loss of projects and aspirations, loss of self as it was before cancer. Other studies of women with MBC have pointed to the pain of a “decline” that affects all spheres of life (Krigel, 2014; Mosher et al., 2013). Our results are in line with the phenomenological work of Marin (2014) for whom the disease often turns out to be a bodily and psychological decay. The image of a collapsed, crushed self provides an experiential description of the vulnerability and inability of the individual to rise above the disease, if he/she is very low. With this in mind, our study suggests that the lived course of the disease, over time, become less combative than passive (Martino et al., 2019), less focused on the fight against cancer than on an attempt to endure the violence of the disease, through a fight against oneself. The image of a fight against oneself and the idea of ending the fight reveal a difficulty in attributing meaning to treatments that prolong life but simultaneously alter its quality. Even in the context of palliative treatments, the women with MBC would face a dilemma: to choose between quantity and quality of life (Ginter, 2020; Lewis et al … 2015). The personal choice to stop the fight by stopping treatments may be a matter of letting go of the disease, or even a refusal to fight the inevitability of death (Sarenmalm et al., 2009). To get rid of the image of war means to get rid of the notion of acting “against” the disease in order to meet the need to “live with” it (Mino et al., 2008). In this regard, our study indicates the desperate need for a medicine that allows patients to maintain a less aggressive relationship with both body and treatments, and also to maintain a more autonomous relationship with those treatments so as to be able to consider oneself both the subject and the actor of one’s life, despite the limitations imposed by the illness (Mino et al., 2008).

Our study indicates that the existential questions of choice, life and its finitude are important to women with MBC because those questions renew their perspective at the heart of their intimate struggle. Indeed, the approach of death can give new meaning to life and the time that remains if it leads women to position themselves more authentically in relation to others and the world (Byock, 2002), whether by making choices and reviewing their priorities (Yalom, 1980), or by transmitting to others the very value of death (Nissim et al., 2012). Our study suggests that this renewed vision comes at the cost of suffering, a result of the violence of this disease. Although deepening one’s ties to the world and others represents a possible source of meaning (Lavoie, 2015; Sarenmalm et al., 2009), our study nonetheless suggests that it is limited by the difficulty in opening up to others about the “negative side” of the disease. By revealing how they restrain their emotions, the bloggers were able to express their attempt to imprison part of their experience within themselves so as not to let the extent of their suffering appear. This finding is in line with Ginter’s study (Ginter, 2020) which showed that women with MBC sometimes use “strategizing disclosure” to gauge the effects that sharing their reality can have on those around them. Many women with MBC fear hurting their loved ones by showing distress, despair or fear (Sarenmalm et al., 2009), and minimal disclosure may be an ethical way to protect them (Vilhauer, 2008). In addition, confrontation with death, loneliness, disembodiment and the search for meaning would all be issues to be disclosed because they can be so hard to express (Best et al., 2015; Mosher et al., 2013). According to Willig (2011), exploring these issues is not socially encouraged, especially if a fight against cancer is not simultaneously engaged to fuel hope and support a positive attitude. Even the blog does not seem exempt from a requirement of positivity. Indeed, the bloggers were often afraid to disappoint or lose readers if they open up too much or dwell too long on their suffering, as if words of anguish or exasperation might discourage readers (De Boer & Slatman, 2014; Sandaunet, 2008).

Despite its limitations, the blog is a unique space to reveal some experiences that cannot be shared elsewhere. The metaphor of the unveiling implies a hidden side of the experience that can be revealed on the blog and integrated into a global experience. Without this unveiling, suffering would remain silent, that is, invisible to others and meaningless for oneself. It is clear to us the bloggers needed to broaden the picture of the cancer experience to expose it in its complexity. The image of the roller coaster illustrated a complex journey made of oscillations and gave visibility to the “low” moments of the disease when the women felt diminished in their being. The oscillatory movement also illustrated the uncertainty about the future, the impossibility of predicting the next lows, future sufferings, or death itself. Indeed, the consciousness of death seemed to appear in fits and starts, reminding the women, in the midst of moments of serenity, how shaky life is. The fact remains that the metaphor of oscillation may ensure coherence to cancer experiences, integrating the ups and downs into a single unit of meaning.

In line with other works (Vilhauer, 2011; Willis et al., 2015), our study suggests that women with MBC suffer from being excluded from public discourse on cancer, which, being focused on survivors, overlook the complexity and seriousness of their experience. By revealing their suffering, the bloggers could expressed their need to be part of the cancer world. On their blogs, they presented two distinct narratives: on the one hand, the dominant narrative of the healing care journey, both visible and pink and, on the other hand, the marginal narrative of the palliative care journey, darker and left in the shadows. The evolution of the bloggers on this last path can be interpreted as a feeling of isolation from the common world as well as a medical abandonment. This isolation adds to the existential isolation everyone feel at the approach of death (Yalom, 1980). Yet unveiling this dark reality on the blog represents a unique opportunity for women with MBC to communicate and commit themselves to the cause of advanced breast cancer. It should be added that expressing their isolation in relation to the dominant narrative could be a way to regain control of their own narrative (Willig, 2011) and to resocialize disease and death (Andersson, 2019; McCosker, 2008).

More broadly, this phenomenological study reveals the importance of two metaphors, the fight and the unveiling, as part of the quest for authenticity and truth at the end of life. As life leaves them, all bloggers were greatly concerned about the disclosure of their unique struggle. They sought to uncover a more authentic representation which better fits their experience. Through this question of the need for authenticity at the end of life, they asked themselves the question of the meaning of Being. Heidegger’s philosophical concept of authenticity provides a better understanding of how women with MBC may experience incurable cancer. Heidegger (1962) suggested that the consciousness of death acted as an incentive to move to a higher mode of existence marked by the “care of Being”. The philosopher distinguished two modes of existence: inauthentic (everyday mode) and authentic (ontological mode) (Yalom, 1980). In the inauthentic mode, the individual gets lost in appearances. The meaning of things remains shrouded in a certain darkness. In the authentic mode, the individual seeks to think things out in depth, notably through reflexive work on his/her freedom to choose, his/her connection to others and the social influences (opinions, words or ideas inherited from others) that constitute him/her but which he/she now tries to appropriate for him/herself (Heidegger, 1962; Yalom, 1980).

The bloggers’ use of the war metaphor can be conceived according to these two modes of existence. Because it is repeated incessantly in social discourse, the war metaphor is today of such common usage that we could associate it with the inauthentic mode. It is then easy to understand why this familiar metaphor were used by the bloggers to express their own particular struggle with cancer. However, it appears to us they move to a higher mode of being when they revisit this warlike metaphor to address it more authentically. Indeed if women with MBC used this language already used by others, they do not apply it as a simple label but rather personalize its meaning. A distance can be established between this war metaphor and oneself, allowing a new meaning to emerge. Women with MBC may integrate their finitude with the feeling of being able to reach a more authentic life by returning to the deeper meaning of their experience, drawing, for example, a fresh vision and renewed energy despite their exhaustion. In this more authentic way, women with MBC may come to “inhabit” their language to better reveal the complex vulnerability of Being, thereby creating a narrative that evades the imperative of a fight at all costs.

### Clinical implications

In oncology, the incurability of the disease and the transition to a palliative approach are often presented to patients using a language loaded with negative meanings, as if future death was senseless or essentially the result of the failure of curative treatments (Kagan, 2017). This way of approaching death can evacuate the significance and opportunities for personal transformations at the end of life. Our phenomenological study calls for a rethinking of this transition to palliative care. Given that the majority of women with MBC have previously been treated for early breast cancer, we must consider the fact that the majority of them will have experienced the transition from curative care—where disease and death were kept away from conversations—to palliative care—where disease is now viewed with humility in the face of life and death (Vachon, 2019). Our study suggests that the boundary between these two distinct cares is porous, and that the transition to palliative care is not necessarily a sign of a change in the way patients cope with the disease: the fight with cancer remains but it is a lost one. Palliative medicine is precisely intended to ensure that death is no longer lived in the mode of defeating the regimen of repair resulting from curative medicine (Castra, 2010, p. 15). It is this crucial objective that the war metaphor undermines. Thus it would be important to reflect upon the role of this metaphor in the continuum of oncological care in order to (1) ensure the application of the values specific to palliative medicine in clinical practice and (2) facilitate the integration of death by patients living with an incurable disease. Similarly, it would be important for clinicians and caregivers to provide a space where the words of patients are valued (Best et al., 2015).

### Methodological considerations

This study present a phenomenological understanding of qualitative evidence. Its quality and value thus do not rely on the notions of objectivity and generalizability but on those of openness, essence and meaning (Dahlberg & Dahlberg, 2019; van Wijngaarden et al., 2017). Consistent with Tracy (2010), we endorse the process of rigour based on sincerity, credibility, resonance, coherence and worthiness. Rooted in IPA, this research gives a rich description of metaphors used by patients with incurable cancer and allows a direct expansion of the existing literature. Moreover, the density and the complexity of the collected blog narratives, the self-reflexivity of the researchers and their sincerity with which they illustrate the data enhance the quality of this study (Tracy, 2010). Furthermore, the bloggers’ evocative writings can meaningfully reverberate and affect the readers who have no direct experience with cancer. It is our hope this research leads to what Tracy (2010) calls resonance and naturalistic generalization.

In this study, transferability is limited by the sample’s homogeneity and the particular context in which understanding emerged. First, we assume that bloggers with MBC had a specific need to share their experience outside the usual framework of discussion with those around them. Second, we assume they may present ideas about themselves that they want readers to see and like, and in this view, our study situates women with MBC in their particular contexts, exploring their personal perspective as it appears on the blog. Thus the data do not give access to a full or total portrait of women’ experience. However, an humanistic approach invites us to “trust” the data (i.e., the women’s words) for what they reveal, not for what they hide. Third, we assume that bloggers with MBC significantly integrated their experience through their effort to create meaning by telling their daily lives on line.

Despite its limitations, this study is the first to our knowledge to explore and understand the incurable breast cancer experience through metaphors. The rich details we have obtained from IPA can give information to clinicians and caregivers on how to better communicate with women with MBC in order to support them. Considering the lack of research on the metaphors of cancer patients, several avenues remain to be explored to better understand the role and potential of creating metaphors in the search for meaning by patients living with a curable or incurable disease, and the role played by clinicians in this search for meaning, knowing that they themselves create metaphors to communicate with patients (Casarett et al., 2010).

## Conclusion

This study revealed two important metaphorical dimensions of storytelling for bloggers with incurable breast cancer: combat and disclosure. The results show the war metaphor had a unique meaning in that context: the bloggers revealed the difficulty of fighting cancer and eventually collapsing in battle, although a renewed look at life had developed parallel to their struggle. By means of those metaphors, the bloggers tried to lift the veil on this complex experience. The results highlight the need for women with metastatic breast cancer to be able to tell and share their story in a supportive context but also the possibility for them to reinvent the war metaphor in order to live in a more authentic way.
